# Origins of specificity during tDCS: anatomical, activity-selective, and input-bias mechanisms

**DOI:** 10.3389/fnhum.2013.00688

**Published:** 2013-10-21

**Authors:** Marom Bikson, Author name, Asif Rahman

**Affiliations:** Department of Biomedical Engineering, The City College of the City University of New YorkNew York, NY, USA

**Keywords:** transcranial direct current stimulation, anatomical brain connectivity, neuromodulation, transcranial magnetic stimulation, stimulation protocol

## Abstract

Transcranial Direct Current Stimulation (tDCS) is investigated for a broad range of neuropsychiatric indications, various rehabilitation applications, and to modulate cognitive performance in diverse tasks. Specificity of tDCS refers broadly to the ability of tDCS to produce precise, as opposed to diffuse, changes in brain function. Practically, specificity of tDCS implies application-specific customization of protocols to maximize desired outcomes and minimize undesired effects. Especially given the simplicity of tDCS and the complexity of brain function, understanding the mechanisms leading to specificity is fundamental to the rational advancement of tDCS. We define the origins of specificity based on *anatomical* and *functional* factors. Anatomical specificity derives from guiding current to targeted brain structures. Functional specificity may derive from either activity-selectivity, where active neuronal networks are preferentially modulated by tDCS, or input-selectivity, where bias is applied to different synaptic inputs. Rational advancement of tDCS may require leveraging all forms of specificity.

## THE NEED FOR tDCS SPECIFICITY

As tDCS is a simple and general technique, applied to a wide range of clinical and cognitive neuroscience applications (Brunoni et al., 2012), a pivotal question to the rational advancement of tDCS is how is specificity achieved? More generally, why does low-intensity direct current produce (desirable) cognitive changes on highly complex tasks recruiting multiple neural pathways or treat multifarious neuropsychiatric disorders (Turkeltaub et al., 2012; Medina et al., 2013; Zimerman et al., 2013)? A question compounded when considering that the DC waveform is does not carry apparent information and that the induced electric field in the brain is low [<1 V/m; (Datta et al., 2009; Ruffini et al., 2012)] producing minimal cell membrane polarization [<1 mV; (Radman et al., 2009)]? Practically, how can stimulation protocols be optimized to promote specificity with the goal of increasing efficacy while reducing undesired side effects? Since these issues are central for the rational advancement of tDCS, here we define both anatomical and functional origins of specificity (Cano et al., 2013). Although task specific effects of tDCS have been shown (Saucedo Marquez et al., 2013; Tang and Hammond, 2013) the mechanistic substrate remains poorly explained.

## ANATOMICAL tDCS SPECIFICITY AND THE “SLIDING-SCALE” MODEL

Anatomical specificity refers to the preferential neuromodulation of targeted brain regions by delivering stimulation current to the targeted area. The number, location, and size of anatomical targets are application specific. For example, the targeted brain region may be a specific cortical area implicated in a task or pathology. Anatomical specificity is achieved only through the control of tDCS electrode dose (defined as electrode montage and current) to guide current to specific brain regions (Peterchev et al., 2011). However, applied without consideration for functional specificity, anatomical specificity is technically and conceptually limited.

Both computational models of current flow in the brain and imaging studies indicate that conventional tDCS methodology using two large sponge pads (5 cm × 5 cm) positioned on the head disperse current through much of the cortex (Datta et al., 2009; Faria et al., 2011; Antal et al., 2012; Neuling et al., 2012) and even deep brain structures (Dasilva et al., 2012). It is important to distinguish between carefully designed studies that demonstrate dose-specific (e.g., electrode position) outcomes (Fiori et al., 2013; Hauser et al., 2013; Penolazzi et al., 2013), from implications that current flow is limited to one brain target. These studies also typically leverage other forms of functional targeting.

Technology for High-Definition tDCS using arrays of electrodes allows categorical increases in anatomical targeting by increasing the focality of current flow (Datta et al., 2009; Dmochowski et al., 2011), but even so, any brain region is evidently involved in multiple tasks. Which presents the inherent conceptual challenge when relying exclusively on anatomical specificity: how can passing DC current through a multi-tasking complex brain region produce specific functional changes?

In the absence of further sophistication, the goals of tDCS are often described as increasing excitability (near the anode) or decreasing excitability (near the cathode) of the target brain region, with brain function and disease thus reduced to a “sliding-scale” of excitability to be adjusted by stimulation (c.f., Rahman et al., 2013). For example, under the sliding-scale concept “anodal tDCS” can enhance the performance of a cognitive task by exciting an implicated brain region. Similarly anodal tDCS is intended to increase left-prefrontal cortex activity in depression and enhance rehabilitation around lesions after stroke. Following early evaluation of Transcranial/transcortical polarization in humans and animal models (Bindman et al., 1962; Redfearn et al., 1964; Elbert et al., 1981), influential neurophysiological studies of tDCS (Nitsche and Paulus, 2000) established modulation of experimental evoked potentials [e.g., motor evoked potential responses to transcranial magnetic stimulation (TMS)]. There is significant extrapolation from these experimental findings to behavior and cognition [TMS evoked responses may provide poor evidence for effects on behavior; (Ridding and Rothwell, 2007)]. Moreover, even the direction of this basic modulation of experimentally evoked potentials is highly sensitive to both tDCS dose [intensity (Matsunaga et al., 2004; Dieckhofer et al., 2006; Batsikadze et al., 2013); direction (Chan and Nicholson, 1986; Bikson et al., 2004; Rahman et al., 2013)] and dependent on brain state (Fröhlich and McCormick, 2010; Reato et al., 2010). Animal studies showing anodal/cathodal DCS producing somatic depolarization/hyperpolarizing (Radman et al., 2009) and increase/decrease in firing rate (Purpura and McMurtry, 1965; Reato et al., 2010), are cited to support a sliding-scale concept, however, global changes in firing rate across a brain region implies a non-specific effect (Reato et al., 2013). In summary, it is reasonable to conclude from neurophysiologic studies that tDCS can produce dose-specific changes in brain functions (Nitsche and Paulus, 2000) that can, with careful extrapolation, serve as a basis for behavioral interventions (Kuo et al., 2013). However, relying only on anatomical specificity by guiding current to specific brain regions (and so the “sliding-scale” rationale) remains limited by the complex and divergent functions of any brain region.

Further sophistication in anatomical targeting follows from considering tangential as well as radial inward/outward currents (Dmochowski et al., 2012) as discussed in a separate article in this special issue. Indeed, the assumption of inward (“excitatory”) and outward (“inhibitory”) current under the anode and cathode, respectively, may be a further over-simplification. Electrophysiological studies in animal models of DC stimulation suggest differential processing of afferent information (Rahman et al., 2013) and that polarity-specific effects invert due to neuronal morphology (Bikson et al., 2004; Kabakov et al., 2012).

## ACTIVITY-SELECTIVITY AND TASK-SPECIFIC MODULATION

Activity-selectivity refers to tDCS preferentially modulating a neuronal network that is already activated, while not modulating separate neuronal network that are inactive. The active neuronal network may be activated for a host of reasons described. The active and inactive networks can in fact overlap in space (e.g., in the same cortical column) such that activity-selectivity does not require physical separation in contrast to anatomical specificity – therefore, we refer to activity-selectivity as a form of functional specificity. The active network may represent a subset of neurons and/or a subset of connections (synapses). Because tDCS produces low-intensity electric fields in the brain, “sub-threshold” neuromodulation may reflect changes in ongoing processes (Reato et al., 2010) in contrast to supra-threshold driven firing by TMS. Activity-selectivity thus assumes there is some feature of the active network that makes it preferentially sensitive to modulation by tDCS compared to other inactive networks. We consider two neurophysiological substrates for this preferred sensitivity: ongoing activity-selectivity and input-selectivity.

Activity-selectivity is based on the assumption that tDCS will preferentially modulate specific forms of ongoing activity. For example, at a cellular level, direct current stimulation (DCS) may enhance plasticity in a given synaptic pathway while stimulated at a preferential frequency (0.1 Hz in Fritsch et al., 2010) or consolidate a specific pattern of activity presented during DCS (Morrell, 1961). DCS may preferentially modulate the level of potentiation in the activated pathway (Ranieri et al., 2012). DCS may facilitate long-term potentiation through membrane polarization and removal of Mg^+^ block (Stagg and Nitsche, 2011) but only those pathways activated during DCS (by a task or experimental stimulation) would benefit from this facilitation. DCS may be too weak and/or unspecific in isolation to enhance synaptic efficacy, but may boost ongoing (e.g., Hebbian) plasticity activated by task performance (i.e., modulation of input specific plasticity along an activated synaptic pathway while sparing quiescent synapses). In humans, transcranial electrical stimulation may also preferentially modulate networks with heightened oscillatory activity (Reato et al., 2010) or preferentially change the progression of an active network during memory consolidation or synaptic downscaling (Reato et al., 2013).

At a behavioral level, specific brain activity is often targeted by training in conjunction with tDCS with the goal that this select activity be sensitized to tDCS neuromodulation (and so implicitly other brain functions not active in training may be less so). For example use-dependent modulation and learning of motor skills is modulated by tDCS (Reis and Fritsch, 2011; Madhavan and Shah, 2012). Clinically, tDCS is often applied to enhance the efficacy of rehabilitation or cognitive training (Edwards et al., 2009; Kuo and Nitsche, 2012; Gomez et al., 2013; Leśniak et al., 2013; Ochi et al., 2013), which may further confer functional specificity through activity-selectivity. Clinically when tDCS is applied to subjects at rest, we can speculate that any functional-specificity results from increased sensitivity of pathological network activity to tDCS (e.g., dysfunctional pain or mood regulating networks). It has been speculated that altered network function associated with brain injury (stroke) may alter the susceptibility to tDCS (Olma et al., 2013). Generally, any interaction between brain activity and the efficacy of tDCS modulation (Kim and Ko, 2013; Pirulli et al., 2013) suggests “tDCS can be highly focal when guided by a behavioral task” (Lapenta et al., 2013).

Although the mechanisms may vary, in any case, functional specificity through activity-selectivity presumes the enhanced activity of the network makes it preferentially sensitive to modulation by tDCS. Thus activity-selectivity necessitates an ongoing network process becoming preferentially tuned to influence by DCS compared to the myriad of other ongoing (background) brain functions.

## INPUT-SELECTIVITY AND BIAS

A third form of specificity we define here is input-selectivity, which assumes a neuronal network that is predisposed to serve at least two functions or operate in at least two states such that tDCS can switch the network from one function/state to another: for example attentional bias in the prefrontal cortex (Eldar et al., 2013). tDCS would change the state of the system toward a different input bias and thus enhance information processing of a specific stream of information. Input-selectivity may activate endogenous “gating” systems (e.g., gate theory of pain) or bi-stable neuronal states – where a non-specific DC signal is able to “switch” a system between complex functions or modes. In contrast to a sliding-scale hypothesis for a stimulated brain region or activity-selectivity affecting a specific ongoing process, input-selectivity implies a regional process is enhanced at the cost of another process – not that input-selectivity results in a zero-sum effect in regards to lasting cognition or behavior outcomes. However, input-selectivity does emphasize the “cost” of acute stimulation. Input-selectivity is also considered a form of functional-specificity since it does not require gross anatomical targeting of current flow. Input-selectivity thus differs conceptually from functional-selectivity (as defined above) in that it does not presuppose co-activation (e.g., by training), and moreover implies that one process may be enhanced at the cost of enhancing another.

Many animal studies that have investigated the modulation of information processing, for example through synaptic efficacy [as opposed to simply membrane polarization and excitability (Chan and Nicholson, 1986; Radman et al., 2007)], have observed that DCS will differentially modulate incoming inputs. We initially showed in hippocampal slice that DCS enhances some and inhibits other afferent inputs (Bikson et al., 2004), a finding verified (Kabakov et al., 2012) and extended to the cortex (Rahman et al., 2013). The cellular origins of bias in favor of selective inputs are twofold. First, although anodal and cathodal tDCS are mistakenly referred to as depolarizing and hyperpolarizing, it is more accurate to describe tDCS as redistributing polarization across the cellular axis, for example one dendritic branch versus another (Fritsch et al., 2010; Rahman et al., 2013). This change in “weights” across the dendrite may provide a cellular substrate to influence the input bias of a network. Second, polarization of afferent axons itself appears to exert pathway specific modulation (Arlotti et al., 2012; Kabakov et al., 2012; Rahman et al., 2013).

Clinically, the concept of input-selectivity can be extended to (selective) attention and working-memory, as well as disease states such as ADHD (Levy, 2004), anxiety, and schizophrenia (Grace, 2000); which are indeed already indications explored for tDCS (Kang et al., 2009; Faber et al., 2012; Demirtas-Tatlidede et al., 2013; Shiozawa et al., 2013). The prefrontal cortex, an anatomical target for several indications including depression (Loo et al., 2012), is indeed implicated in executive function and differentiating among (conflicting) inputs. The concept of bias-selection is consistent with stimulation inhibiting some functions while enhancing others within any given region (Iuculano and Kadosh, 2013; Tang and Hammond, 2013) as well as modulation of relative value judgments (Vanderhasselt et al., 2013; Votinov et al., 2013), which can be considered as a form of weighting inputs so specific outcomes can be biased.

Future studies on the actions of input-selectivity using tDCS can explore applications of multimodal imaging technologies, including magnetic resonance spectroscopy (MRS), functional magnetic resonance imaging (fMRI), magneto-encephalogram (MEG), and electro-encephalogram (EEG), to further establish the different functional brain-states of a cortical region activated during tDCS (Soekadar et al., 2013). The topic of multimodal imaging in tDCS is discussed further in another article in this special issue of Frontiers in Human Neuroscience (Hunter et al., 2013).

## OUTLOOK FOR tDCS

Anatomical specificity and functional specificity, through either ongoing activity-selectivity or input-selectivity, are not exclusive and may potentially be leveraged together in the development of rational tDCS protocols. In general, we propose that understanding the basis for tDCS selectivity is essential. Although we have focused our discussion to tDCS, the approaches described here would apply to other brain stimulation techniques including DBS, VNS, TMS, tRNS, and tACS (discussed further in another article in this special issue, Reato et al., 2013) as well as ultrasound and light based approaches. But the diversity of applications already investigated for tDCS, including increasing dosage (e.g., weeks of sessions), broader populations (e.g., children), suggests a need to address the basis of specificity to be especially acute. Both time-dependent and homeostatic effects (Penolazzi et al., 2013; Peters et al., 2013) increase the subtlety in tDCS protocol design that may require understanding origins of specificity.

## Conflict of Interest Statement

The authors declare that the research was conducted in the absence of any commercial or financial relationships that could be construed as a potential conflict of interest.
